# Exogenous RNAs for Gene Regulation and Plant Resistance

**DOI:** 10.3390/ijms20092282

**Published:** 2019-05-08

**Authors:** Alexandra S. Dubrovina, Konstantin V. Kiselev

**Affiliations:** 1 Laboratory of Biotechnology, Federal Scientific Center of the East Asia Terrestrial Biodiversity, Far Eastern Branch of the Russian Academy of Sciences, 690022 Vladivostok, Russia; kiselev@biosoil.ru; 2 The School of Natural Sciences, Far Eastern Federal University, 690090 Vladivostok, Russia

**Keywords:** exogenous RNAs, external application, dsRNAs, hpRNAs and siRNAs, RNA spraying, plant gene regulation, gene silencing, RNA interference, plant resistance

## Abstract

Recent investigations documented that plants can uptake and process externally applied double-stranded RNAs (dsRNAs), hairpin RNAs (hpRNAs), and small interfering RNAs (siRNAs) designed to silence important genes of plant pathogenic viruses, fungi, or insects. The exogenously applied RNAs spread locally and systemically, move into the pathogens, and induce RNA interference-mediated plant pathogen resistance. Recent findings also provided examples of plant transgene and endogene post-transcriptional down-regulation by complementary dsRNAs or siRNAs applied onto the plant surfaces. Understanding the plant perception and processing of exogenous RNAs could result in the development of novel biotechnological approaches for crop protection. This review summarizes and discusses the emerging studies reporting on exogenous RNA applications for down-regulation of essential fungal and insect genes, targeting of plant viruses, or suppression of plant transgenes and endogenes for increased resistance and changed phenotypes. We also analyze the current understanding of dsRNA uptake mechanisms and dsRNA stability in plant environments.

## 1. Introduction

Current practices to protect plants and improve crop productivity rely on chemical treatments, which are hazardous to the environment and exert deleterious effects on human health. Constructing transgenic plants is a promising and cost-effective alternative to the application of chemicals. However, there is insufficient information on the consequences of genome modifications, and this raises public concerns on the safety of genetically-modified organisms [1]. There are legislative limitations on the cultivation of transgenic plants in many countries [2]. Thus, the development of new sustainable and environmentally friendly approaches to improve plant qualities without genome modifications is an urgent task.

Currently, multiple investigations show that it is possible to down-regulate the expression of particular genes for the control of pathogen resistance, abiotic stress tolerance, growth processes, and other plant properties via RNA interference (RNAi or gene silencing) induction [3,4]. However, the application of this approach requires double-stranded RNA (dsRNA) delivery into plant cells via the production of transgenic plants or the application of weakened plant viruses [4], which poses technical and safety challenges. Several studies reported on using alternative tools for siRNA and dsRNA delivery into plant cells, such as a nanosecond pulsed laser-induced stress wave or conjugated polymer nanoparticles for siRNA [5,6], or using cell-penetrating peptides or an airgun bombardment device for dsRNA delivery [7,8].

**Figure 1:**
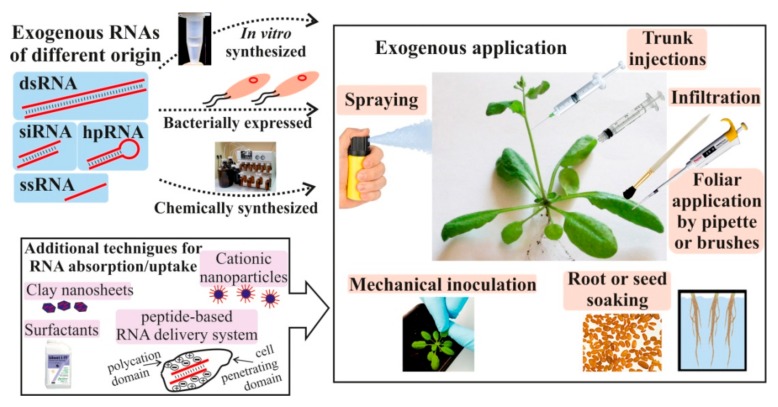
Schematic representation of the type (double-stranded RNA (dsRNA), small interfering RNA (siRNA), hairpin RNA (hpRNA), and single-stranded RNA (ssRNA)) and origin (in vitro, chemically, or bacterially synthesized) of externally applied RNAs and the available RNA application methods (spraying, mechanical inoculation, root or seed soaking, foliar application by pipette or brushes, infiltration, trunk injections) used for RNA delivery in plants. The artificial RNAs could be exogenously applied onto the plant surface using additional techniques to increase the RNA absorption/uptake (cationic nanoparticles, clay nanosheets, surfactants, peptide-based RNA delivery systems).

Recent studies reported on the substantial induction of plant viral [9,10,11,12], fungal [13,14,15,16,17,18], and insect [19,20,21,22] resistance after external application of in vitro synthesized or bacterially produced long dsRNAs, hairpin RNAs (hpRNAs), or small interfering RNAs (siRNAs) targeting essential genes of the plant pathogens and pests. The RNAs were directly applied onto the plant leaves by spray application, mechanical inoculation, loading on clay nanosheets, use of materials promoting RNA adhesion, spreading by pipette or brushes, and delivery by root soaking (Figure 1). Recent studies by Dalakouras et al. [23] and Ghosh et al. [22] also reported on the efficient delivery of exogenous hpRNAs and siRNAs into woody and herbaceous plants by trunk injections, soil/root drench, and petiole absorption. Several reports also provided evidence on the down-regulation of plant transgenes [10,24,25,26] and endogenes [20,27,28] by the naked RNAs or RNAs in a complex with nanoparticles or a protein carrier.

A number of studies provided evidence that the exogenous dsRNAs spread across plant tissues and were processed into siRNAs, leading to the induction of RNAi-mediated silencing of the targeted genes [9,10,11,13,17,26]. RNAi is a natural regulatory and defense mechanism that is implicated in the control of the plant response to pathogens or other environmental stresses, growth, and development [29,30,31]. RNAi is known to mediate the plant response to undesirable nucleic acids, and transposon and transgene activity, and is involved in the control of the expression of endogenous protein-coding genes. Long dsRNAs and hpRNAs are key players in RNAi induction, being recognized and processed into siRNAs, micro RNAs, or other small RNAs, by DICER-like ribonucleases (DICER) [32,33]. The resulting small RNAs are then incorporated in the RNA-induced silencing complex (RISC) that guides sequence-specific degradation or translational repression of homologous mRNA targets. In plants, the RNA silencing signal is known to move short distances from cell to cell (most likely via intercellular plasmodesmata) or long distances (systemically via the phloem vasculature) [34,35]. Although there is limited information regarding the nature of the mobile silencing signal, a number of reports provide strong evidence that the signal is a nucleic acid, most likely 21 to 24 nt siRNA and miRNAs, acting as mobile silencing signals in plants [34,36,37]. However, there is scarce and inconsistent data on the mobile form of the RNAs (double or single stranded, naked, or in a complex with proteins) [34].

**Figure 2:**
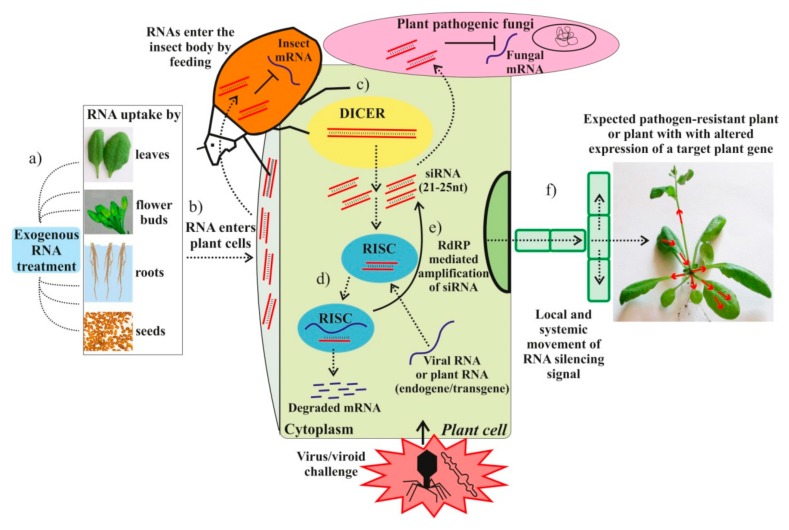
Schematic representation of exogenous RNA applications for RNA interference (RNAi) induction and degradation of the target plant pathogen or endogenous plant mRNAs. (**a**) Exogenous artificial RNA provided in a solution and applied onto plant leaves, flower buds, roots, or seeds. (**b**) The exogenous RNAs are taken up and transported into the cytoplasm via an undefined mechanism. (**c**) The dsRNA or hpRNA molecules are recognized by a ribonuclease, DICER-like (DICER), which cleaves the dsRNA into siRNAs. (**d**) The siRNAs are then incorporated in the RNA-induced silencing complex (RISC) that guides sequence-specific degradation or translational repression of homologous mRNAs. (**e**) The components of the siRNA/mRNA complex can be amplified into secondary siRNAs by the action of RNA-dependent RNA-polymerase (RdRP). (**f**) Movement of the RNA silencing signal between plant cells and through the vasculature. Dashed arrows depict different steps of the RNAi induction process and dsRNA/siRNA movement between plant cells and plant pathogens. The solid arrow depicts the RdRP-mediated amplification of siRNA. Red arrows depict the local and systemic movement of the RNA silencing signal in the plant.

The available studies show that the exogenously applied RNA molecules are capable of affecting the mRNA levels of target genes in the plant genome or in plant pathogens and are promising agents for regulating plant properties (Figure 2). Active studies in this direction are needed to shed light on the plant’s RNA perception and to promote the development of new innovative approaches in plant biotechnology. In this review, we summarize and discuss the findings to date on the applications of exogenous RNAs onto plant surfaces for post-transcriptional regulation of genes in the plant genome and genes of infecting pathogens. This review also focused on the current understanding of extracelluar dsRNA stability, recognition, and uptake mechanisms.

## 2. Induction of Plant Virus Resistance by Foliar-Applied dsRNAs, hpRNAs, and siRNAs

Induction of RNAi or gene silencing is known as a conserved regulatory mechanism, playing important roles in plant viral defense induction [38]. The viral dsRNAs or hpRNAs formed as the replication and/or transcription intermediates of invading DNA and RNA viruses are processed into siRNAs by the plant host RNAi machinery and then direct degradation of the complementary viral RNAs or methylation of viral DNAs. Intensive targeting of the viral RNA and DNA by the RNAi machinery eventually slows down or terminates virus accumulation.

Several experimental studies analyzed the effects of exogenous application of virus-derived dsRNAs or hpRNAs on the resistance to these viruses of various plant species, such as tobacco, tomatos, maize, papayas, and orchids (Table 1). Plants were treated either with in vitro-synthesized RNA preparations or by crude nucleic acid extracts purified from dsRNA/hpRNA-expressing bacterial strains (Table 1). The crude bacterial extracts were treated with DNAse and RNAse before application in most cases. The RNase III deficient *Escherichia coli* strains, HT115, M-JM109, or M-JM109lacY, were utilized for efficient and stable in vivo production of large amounts of dsRNA. Recently, Niehl et al. [39] developed a stable and efficient in vivo dsRNA production system in *Pseudomonas syringae* bacteria. These studies have shown that foliar application of in vitro-synthesized or bacterially produced dsRNAs or hpRNAs targeting viral genes, such as replicase (*RP*) or coat protein (*CP*) genes, delayed the onset of viral infection symptoms, reduced the infection symptoms and the number of infected plants, lowered viral titer, and decreased transcription levels of the targeted viral genes. Furthermore, there were reports showing that siRNAs [40] and single-stranded RNA (ssRNAs) [41] induced viral resistance, although the antiviral effects were lower than that of dsRNAs. However, Tenllado and Díaz-Ruíz [42] reported that the application of both sense and antisense ssRNAs did not interfere with the virus infection. The available studies demonstrated that plant treatments with external virus-derived RNAs interfered with the viral infection and elicited plant resistance against the invading viruses. The results summarized in Table 1 indicate that the antiviral effect of external virus-related RNAs was sequence specific, since the protection induction was not observed when nonviral RNAs or RNAs of nonrelated viruses were applied.

The investigations documented that the dsRNAs/hpRNAs-induced protective effects were maintained for at least 20 to 70 days post virus inoculation (Table 1). This effect maintenance period is related either to the complete absence or to the development of mild viral infection symptoms after virus inoculation. Only several studies (Table 1) tested different times of virus inoculation after the RNA treatments to determine the duration of the protection period, in which further virus inoculations would be ineffective, and most of these studies demonstrated that the RNA protective effects were lower or not observed when the virus was inoculated 2 to 7 days after the RNA application [10,12,39,43,44,45]. It is possible that the instability of the naked RNAs applied onto the plant surface could account for the short period of virus protection. In a recent study, Mitter et al. [10] reported that loading the virus-specific dsRNA on layered double hydroxide (LDH) clay nanosheets provided higher stability to the dsRNA and helped overcome the problem of a short RNA protection window. According to the report, delivery of the dsRNA loaded on the clay nanosheets by a single spray extended protection against further virus inoculations from 5 to 7 days to at least 20 days. Most available investigations reported on mechanical co-inoculation of the RNA and viruses by gently rubbing the plant leaf surface with the inoculum using carborundum as an abrasive (Table 1). Celite powder was also used as an abrasive in two studies [39,41]. Also, there were studies where the dsRNA or hpRNA were sprayed on the plant surface using an atomizer or a clean perfume dispenser [39,43,44]. Length of the applied dsRNAs ranged from 237 bp to 2 kb (Table 1). Tenllado and Díaz-Ruíz [42] compared the efficiency of dsRNA of different lengths and demonstrated that smaller dsRNAs had a much lower antiviral effect than dsRNAs encoding the major part of the targeted virus gene, and therefore the dsRNA protection effect could be length-dependent. It is possible that the mode of RNA application, optimization of the RNA amount, or RNA length variations could extend the period of protection and increase the efficiency of the plant’s antiviral defense.

Such methods as Northern blotting, stem-loop PCR, or deep RNA sequencing suggested that the exogenously applied dsRNA are processed to the viral-derived siRNAs and initiate viral RNA silencing in treated plants, supporting the conclusion that observed virus resistance is indeed an RNAi-mediated process [9,10,11,46]. Viral-derived dsRNAs [9,10] and siRNAs [11] have been shown to spread systemically from treated leaves to non-treated ones. However, Tenllado and Díaz-Ruíz [42] did not observe the presence of exogenously applied dsRNA in non-treated leaves. 

## 3. Plant Treatments with dsRNA for Insect Pest Resistance

In the past few years, multiple studies showed that dsRNAs or hpRNAs complementary to some important genes of insect pests can mediate RNAi-mediated post-transcriptional down-regulation of the genes and lead to increased insect mortality, reduced growth and fecundity, and lowered insecticide resistance [47]. The RNAi induction was achieved by several approaches, including generation of transgenic plants expressing a particular dsRNA construct [47,48], microinjections of in vitro synthesized dsRNA into insect pests [49,50], or the feeding of insect pests with dsRNAs [48,50,51]. There were also studies showing that external application of dsRNA to insect pests and their larvae can mediate silencing of the targeted insect genes and increase insect mortality due to the penetration of dsRNA through the insect cuticle [52,53].

In addition, it has been documented that plants naturally produce endogenous dsRNAs and accumulate dsRNA-derived sRNAs [51,54]. Ivashuta et al. [51] have shown that western corn rootworm and the Colorado potato beetle uptake the plant endogenous dsRNAs and siRNAs through ingestion of plant specimens. Subsequently, several encouraging reports summarized in Table 2 indicated that foliar application of in vitro synthesized dsRNAs led to dsRNA movement into the plants/insects and lowered insect biological activity (weight, development, or mortality). According to Li et al. [20], rice and maize root soaking in dsRNA-containing solution led to dsRNA absorption and increased insect mortality. San Miguel and Scott [19] demonstrated that the biological activity of the Colorado potato beetle was considerably lowered when applying *Actin*-dsRNAs of different lengths on potato leaves, with the most severe effects observed for longer dsRNAs. Consequently, insect consumption of dsRNA-treated plant foliage was greatly reduced. Notably, plants treated with dsRNA several weeks before larvae feeding assays were as effective as plants treated shortly before insect feeding. However, the study also showed that the insect-protective effect did not extend to untreated potato leaves, since only larvae held on treated leaves were affected. In contrast, Gogoi et al. [21] demonstrated that foliar-applied dsRNAs were detected both in treated and systemic tomato leaves, although the insect biological activity was not analyzed. Gogoi et al. [21] have shown that external dsRNAs were taken up by different agricultural pests, including aphids, whiteflies, and mites, that fed on either treated or systemic tomato leaves. Ghosh et al. [22] demonstrated that transcript levels of targeted *JHAMT* and *Vg* genes were down-regulated by exogenously applied synthetic dsRNAs in brown marmorated stink bugs feeding on the common bean. Ghosh et al. [22] provided several dsRNA delivery methods, including spraying, soil/root drench application, and trunk injections for citrange plants, and reported that the applied dsRNAs were detected in the treated plants for 7 weeks after a single exposure [22,55]. In summary, there are now encouraging, but sparse studies, where both dsRNA movements and insect biological activity have been analyzed after foliar application of dsRNA on the plant surface.

## 4. Induction of Plant Fungal Resistance by Foliar-Applied dsRNAs and siRNAs

Recent findings indicated that small RNAs molecules derived from plant fungal pathogens can translocate into plant host cells and silence certain plant immunity genes, assisting suppression of plant immunity and promoting fungal infection [56]. Conversely, it has been shown that sRNAs of plant hosts can be delivered into fungal pathogens in exosome-like extracellular vesicles, accumulating at the infection sites to induce RNAi and silence essential fungal genes important for pathogenicity, and inhibit the infection [14,57]. Fungal pathogens have also been shown to take up not only external small RNAs, but also dsRNAs [14], suggesting that dsRNAs could also undergo such cross-kingdom trafficking. The discovery and further investigation of this natural RNA trafficking mechanism may help develop new approaches for the delivery of pathogen-targeting RNAs and for the control of plant fungal infections. The process of RNA trafficking from plant hosts to interacting pathogens was termed host-induced gene silencing (HIGS) and was extensively exploited in recent years as a method of crop protection and disease control by transforming plants with dsRNA constructs, targeting essential pathogen genes [58,59].

Recently, a number of studies demonstrated that the application of in vitro synthesized dsRNAs or siRNAs targeting essential fungal genes onto the plant leaf surface attenuated fungal infection by inhibiting fungal growth, altering fungal morphology and reducing pathogenicity, and led to the development of weaker plant disease symptoms (Table 3). Exogenous spray application of dsRNAs to the plant surface, termed spray-induced gene silencing (SIGS), is currently considered as an innovative strategy for crop protection from fungal diseases [59,60]. In addition, foliar application of dsRNAs complementary to the genes essential for fungicide resistance lowered the fungicide resistance of the infecting fungal pathogens [17,18].

Two studies demonstrated that the foliar-applied dsRNAs entered not only fungal cells, but also plant cells and the plant vascular system, where they were processed into siRNAs and induced RNAi-mediated silencing of the targeted fungal genes [13,17]. Fungal growth was inhibited both in the directly sprayed and non-sprayed tissues, leading to the induction of local and system fungal resistance. Two pathways were proposed for the dsRNAs and siRNAs applied onto the plant surface to enter fungal cells: (1) The foliar-applied RNAs enter fungal cells directly and induce fungal RNAi machinery; and (2) the RNAs are taken up by plants first, induce plant RNAi machinery, and then translocate to the fungal cells [13,14,17]. Song et al. [17] studied the two possible pathways in more detail and found that the *Myo5* gene silencing effect in *Fusarium asiaticum* was maintained only if the dsRNA was continuously supplied, since *F. asiaticum* was not capable of maintaining siRNA amplification. In plant cells, however, the siRNAs derived from the *Myo-5* dsRNA were detected up to 8 days after fungal inoculation without a continuous supply of the dsRNA. The results by Song et al. [17] indicated that the foliar-applied *Myo5*-dsRNAs were processed into siRNAs and then amplified by RNA-dependent RNA polymerases (RdRP), leading to secondary siRNA formation. According to Song et al. [17], the dsRNA was absorbed more efficiently via the wounded surface of the tip cut wheat coleoptiles than via the intact surface.

Most of the available studies on fungi exploited a simple spray or drop application of the RNA solutions before fungal inoculation, which was performed from 12 hpt to 5 dpt (Table 3). Silwet L-77, a non-ionic surfactant, was used as a wetting agent in only one of the studies applying external dsRNA to affect the fungal growth and plant disease resistance (Table 3; [15]). Thus, little is known on the possible RNA application modes, various RNA stabilizing agents, or RNA uptake promoting factors. Further investigation and detailed comparisons of RNA application conditions are necessary. In addition, there are no systematic investigations showing the effect of different fungal inoculation times on the duration of dsRNA-induced antifungal protection and the duration of the protective effect. 

## 5. Silencing of Plant Endogenous Genes and Transgenes via dsRNA and siRNA Application

Since dsRNA molecules can be taken up and spread across plant tissues, where they turn into siRNAs, active studies for plant gene regulation via exogenous RNA application are urgently required to influence plant properties, including plant disease resistance and other traits. Confirmation and detailed investigation of the mechanisms responsible for the uptake of external RNAs, their processing, and silencing induction are required for further development of plant molecular biology and biotechnology.

Plant transgenes represent a good model for the studies aimed to affect the expression of specific plant genes due to higher transgene sensitivity for silencing, clearer transgene silencing effects, and a lower likelihood of secondary effects in comparison with the silencing of plant endogenous genes. Currently, there is compelling evidence showing that plant transgenes are more sensitive to transcriptional and post-transcriptional silencing than endogenes due to the absence of introns and 5′ and 3′ untranslated regions (UTRs), which are known to suppress RNA silencing, and to a higher production of aberrant mRNAs transcribed into secondary dsRNAs [61,62,63,64,65,66]. Therefore, it is reasonable to analyze the effect of exogenous RNAs on plant transgene transcript levels first and to search for the most optimal conditions for transgene silencing. To date, several studies reported on plant transgene regulation by foliar applications of dsRNAs and/or siRNAs (Table 4). According to two reports, direct application of naked transgene-encoding siRNAs (e.g., by wiping, spraying, injection) did not affect the fluorescence of yellow fluorescent protein (YFP) and green fluorescent protein (GFP) in transgenic tobacco and *Arabidopsis* plants without additional techniques, including use of a protein carrier [24] or high-pressure spraying [25]. Using confocal microscopy and Western blotting, Numata et al. [24] demonstrated that infiltration of *Arabidopsis* and poplar with *YFP*-encoding siRNA duplexes in a complex with a carrier peptide, (KH)9-Bp100, reduced YFP fluorescence and YFP protein levels. According to Dalakouras et al. [25], high-pressure spraying of tobacco plants with GFP-siRNAs using a conventional compressor and an air brush pistol lowered GFP fluorescence. There were also two studies indicating that direct application of transgene-encoding long dsRNAs suppressed transgene expression. Mitter et al. [10] reported that the application of both naked *GUS*-dsRNA and *GUS*-dsRNA loaded on clay nanosheets to *Arabidopsis* considerably lowered GUS activity. Foliar application of naked *NPTII*- and *EGFP*-encoding dsRNAs [26] to four-week-old *Arabidopsis* plants by spreading with individual soft brushes suppressed *NPTII* and *EGFP* transgene mRNA levels, lowered EGFP fluorescence and protein levels, increased *EGFP* and *NPTII* DNA methylation, and led to *EGFP*-siRNA detection [26]. It has been shown that read-through transgene transcripts that included both *NPTII* and *EGFP* coding regions and their regulatory regions were generated [26]. The results obtained suggested that the silencing of transitivity and secondary siRNA formation occurred before and after the treatments in the transgenic *A. thaliana* plants and were enhanced after the dsRNA treatments. In a separate study, synthetic *NPTII*-encoding siRNAs were applied, leading to a down-regulation of the targeted transgene transcript levels [67].

Furthermore, the existing literature provides several examples of plant endogene regulation by exogenously applied dsRNAs (Table 4). First, the patent by Sammons et al. [68] established the possibility of down-regulating mRNA transcript levels of plant genes encoding for herbicide resistance using the foliar application of naked dsRNA, siRNA, ssRNA, or even DNA molecules. Then, Lau et al. [28] showed that the direct application of crude bacterial extract containing *MYB1*-dsRNAs onto flower buds of the orchid, *Dendrobium hybrid*, suppressed expression of the target *DhMyb1* gene and changed the phenotype of floral cells (from conical to flattened epidermal cells). According to Li et al. [20], plant root soaking into dsRNA containing water solutions led to dsRNA absorption and suppressed expression of the targeted plant *Mob1A*, *WRKY23*, and *Actin* genes, and elevated transcript levels of *Ago* and *DICER* genes, which are key RNAi players. These findings suggested that the absorbed dsRNA can trigger plant RNAi. There were two studies where external siRNA or dsRNA application influenced the plant phenotype, being applied in a complex with a peptide carrier [24] or nanoparticles [27]. Infiltration of *Arabidopsis* leaves with siRNAs targeting the chalcone synthase (*CHS*) gene in a complex with a peptide carrier induced a local loss of anthocyanin pigmentation, but the mRNA or protein levels of CHS were not analyzed [24]. Jiang et al. [27] have shown that the application of dsRNA mixed with cationic fluorescent nanoparticles G2 suppressed expression of *SHOOT MERISTEMLESS* (*STM*) and *WEREWOLF* (*WER*) genes implicated in shoot apical meristem regulation and root epidermis control in the seedling of *A. thaliana*. The G2/dsRNA-treated plants exhibited retarded growth and reduced meristem size, while treatment with only dsRNA did not lead to these effects.

## 6. Stability of dsRNA in the Environment

It is possible that the instability of the naked dsRNAs and siRNAs applied onto the plant surface could account to the short period of effect duration, which was documented for virus targeting. To date, little is known about the fate of plant surface-applied dsRNAs, hpRNAs, and siRNAs as well as on the rate of their biodegradation in various environments. According to Karan et al. [69], viral dsRNA was highly stable (up to 6 months) when infected plant leaves were dried at high or low temperatures and stored at room temperature. An analysis of the fate of soil-applied corn rootworm-related *DvSnf7*-dsRNA in three types of soil revealed the relative instability of the dsRNA in the soil environment [70]. The authors reported that the dsRNA was degraded and was non-detectable in soil 48 h after fortification regardless of the soil type and properties. In a recent study, Parker et al. [71] investigated the adsorption and degradation of dsRNA in agricultural soils using a novel experimental approach based on phosphorus-32 (32P)-radiolabeled *GFP*-dsRNA. The results indicated that the dsRNA was both adsorbed on soil particles across soils and gradually degraded by extracellular microbial hydrolases in the soil solutions. The intact dsRNA was no longer detectable after incubation for 24 h in soil solutions. In addition, Parker et al. [71] also reported on the potential uptake and utilization of the dsRNA by microorganisms. Their findings also indicated that incubation from 0.5 h to 24 h resulted in a substantial transfer of the labeled dsRNA molecules or their parts into microbial cells or on suspended particles in soil solution. Fisher et al. [72] determined the fate of *DvSnf7*-dsRNA following an over-water application using aquatic systems containing natural water and sediments. The dsRNAs were almost dissipated in the water systems and were undetectable within 4 to 7 days. However, Li et al. [20] reported on a relative dsRNA stability (up to 8 h) under a continuous 30 to 50 °C temperature and ultraviolet irradiation. According to Mitter et al. [10], foliar-applied virus-derived dsRNA was almost undetectable 20 days after spraying. In contrast, San Miguel and Scott [19] showed that *Actin*-dsRNA of the Colorado potato beetle, directly applied to potato leaves, was stable for at least 28 days under greenhouse conditions. The authors reported that the dsRNA was not readily removed with water after drying.

To our knowledge, there are no other detailed studies determining the stability of dsRNA or small RNAs applied to different plant environments or onto plant surfaces. In our recent investigation, considerable amounts of foliar-applied transgene-encoding dsRNAs were detectable in RNA probes purified from treated *Arabidopsis* leaves 1 and 7 days post treatment, while amounts of dsRNA sharply dropped 14 days post-treatment [26]. Further studies are needed to provide thorough knowledge of dsRNA and small RNA availability and stability when applied onto plant surfaces or after soil/water treatments. Most of the studies summarized in Table 1, Table 2, Table 3 and Table 4 utilized non-modified RNAs dissolved in water. There are multiple studies on mammalian systems showing that siRNA modifications, such as 2′-methoxy (2′-OMe), 2′-*O*-benzyl or 2′-fluoro (2′-F), increased siRNA stability and potency without compromising or modifying the silencing effect [73]. It is possible that RNA modifications, by mixing the RNAs with stabilizing agents or surfactants, could extend their half-lives and effective periods.

## 7. Plant Nucleic Acid Recognition and Uptake

A number of studies provided evidence for the uptake of naked dsRNAs into plant cells and the plant vascular system through the leaf surface, leading to RNAi induction in the plant and/or the fungal pathogen [10,13,17]. Foliar-applied dsRNAs were detected in the xylem of barley leaves, apoplast and symplast of ploem parenchyma cells, companion cells, and mesophyll cells, as well as in trichomes and stomata [13]. According to Mitter et al. [10], both naked synthetic viral dsRNA and viral dsRNA loaded on clay nanosheets were taken up into the xylem of *Arabidopsis* leaves. In addition, dsRNA loaded on clay nanosheets showed uptake in the spongy mesophyll. Song et al. [17] analyzed whether labeled dsRNA was taken up by wheat cells directly or via the wounded surface of wheat coleoptiles and found that the dsRNA was absorbed more efficiently via the wounded surface. According to microscopic studies, dsRNA is primarily located in the local tissue of the cut surface, in the lignified tracheary elements of the distal tissue, and in the parenchyma cells around the tracheary elements. The authors proposed that the used dsRNA entered the damaged cells of the wounded coleoptile surface and was transferred via the tracheary elements. Detection of the fluorescence signal in the wheat tissues suggested that the dsRNA was stable in the plant tissue for at least up to 8 days. Overall, there is scarce information on the stability of exogenous dsRNA/siRNA taken up within plant cells and tissues. Koch et al. [13] reported on the fluorescent signal detection of labeled dsRNA in barley vascular tissue 24 h after spraying. Faustinelli et al. [74] demonstrated that exogenously applied synthetic 27 nt siRNAs spread systemically and were stable for at least 30 days in in vitro peanut plants. In addition, there are several studies reporting on the stability of dsRNA applied onto plant leaf surfaces and showing that dried naked dsRNA or dsRNA loaded into LDH nanosheets were stable on plant surfaces for up to 30 days [10,19].

It is likely that externally applied synthetic dsRNAs and siRNAs (Table 1, Table 2, Table 3 and Table 4) could enter plant tissues and cells, exploiting the same natural mechanisms as extracellular nucleic acids originating from plant microbial pathogens, insects, or viruses. The existing literature provides limited information on the natural mechanisms ensuring recognition, uptake, and translocation of exogenous nucleic acids in plant tissues. Paungfoo-Lonhienne et al. [75] demonstrated that labeled external phosphorothioate DNA oligonucleotides of 25 bp were taken up by plant root hairs and pollen, used as a phosphorus source, and stimulated root and pollen tube growth. Pathogenesis-related extracellular DNA and RNA derived from plant microbial and virus pathogens are being considered to induce plant innate immunity and regulate self- and non-self-recognition in plants [76]. It is proposed that extracellular pathogen- and virus-related DNA and RNA molecules are perceived as genuine microbe- and pathogen-associated molecular patterns (MAMPs and PAMPs) in plants and act via pattern recognition receptors (PRRs) to induce pattern-triggered plant immune (PTI) signaling [76]. External application of bacterial DNA [77], intact bacterial RNAs [78], and virus dsRNAs [79] to *Arabidopsis* and tobacco plants activated PTI and provided reduced disease symptoms upon infection with pathogens. External nucleic acid applications induced signaling events, such as mitogen activated protein kinase (MPK) activation, reactive oxygen species (ROS) production, or defense gene expression, were similar to that of typical MAMP and PAMP downstream signaling. Recently, Duran-Flores and Heil [80] hypothesized and provided evidence that extracellular self-DNA acts as a damage-associated molecular pattern (DAMP) in common bean plants and is implicated in self- versus non-self-recognition. The external application of fragmented self-DNA induced immunity responses and defense against bacterial pathogen and herbivores.

Thus, there is scarce information on the molecular mechanisms of nucleic acid recognition and uptake by plants. To date, no specific receptors responsible for the recognition and uptake of extracellular DNA have been identified in plants. First evidence from the study by Niehl et al. [79] indicated that the PTI mediated by external RNAs was independent of RNA silencing processes. Exogenously applied virus-related dsRNA induced PTI responses in *Arabidopsis* via a somatic embryogenesis receptor-like kinase 1 (SERK1), and not through antiviral DICER-like proteins (DCL) [79]. This study suggested SERK1 as a potential dsRNA receptor. Further research is needed to clarify whether and how extracellular nucleic acids, which are recognized as MAMPs and PAMPs, are taken up and processed in plants.

## 8. Conclusions and Future Perspectives

Global growth of the human population, reductions of farmland, climatic change, and concerns about the safety of genetically engineered plants has promoted the development of new non-transgenic and environmentally-friendly approaches to regulate plant disease resistance and other plant properties. Application of RNAs onto plant surfaces could also help circumvent the absence of efficient and rapid transformation protocols for many agronomically important plant species.

Although the exact mechanism of external RNA recognition, uptake, and transport remains to be determined, recent studies demonstrated that exogenous RNA application is a promising strategy for the regulation of plant properties and requires further research (Table 1, Table 2, Table 3 and Table 4; Figure 1 and Figure 2). New knowledge about RNA stability in the environment, plant perception of external RNAs, and the mechanisms governing cross-kingdom RNA trafficking between plants and invading pathogens could expand our understanding of host–pathogen interactions and disease control. The present analysis also highlights the need to pursue systematic investigations not only for further documentation and investigation of the effects from exogenous RNA applications, but also for the search and optimization of the most optimal conditions for RNAi induction and the targeting of gene down-regulation. It is possible that a number of factors, including RNA concentration, RNA length, treated plant surface, application techniques, plant age, soil humidity, RNA modifications, or even time of day, are important criteria that can influence the efficiency of silencing. Furthermore, the application of additional agents, e.g., nanoparticles, RNA stabilizing agents, materials promoting RNA adhesion, or protein carriers, could assist the development of new approaches in biotechnology involving exogenous RNA treatments.

## Figures and Tables

**Table 1:** Foliar application of RNAs for plant virus resistance.

Target	RNA Type, Size and Origin	RNA Amount	RNA and Virus Application	Plant Host	Effect	Effect Maintenance	Reference
*RP* gene of PMMoV, TEV, and AMV	In vitro synthesized dsRNA (PMMoV 315, 596, and 977 bp; TEV 1483 bp; AMV 1124 bp)	5 µL of each dsRNA (2.5 µM)	Mechanical inoculation (virus co-inoculation)	Tobacco, pepper	Resistance to PMMoV, TEV, and AMV (assessed at 5–7 dpi)	Up to 21 dpi	Tenllado and Díaz-Ruíz (2001) [42]
*RP* gene of PMMoV	Crude extracts of bacterially expressed dsRNA (977 bp)	10 µL of bacterial extract (1.5–3 µg/µL)	Mechanical inoculation or spraying with atomizer (virus co-inoculation or 1, 3, 5, and 7 dpt)	Tobacco	Resistance to PMMoV (assessed at 7 dpi, 30 dpi)	Up to 70 dpi	Tenllado et al. (2003) [43]
Viroid-specific dsRNAs	In vitro synthesized dsRNA and siRNA (less-than-full-length)	1250 to 5000 molar excess of dsRNA; 100 molar excess of sRNA over viroid RNA	Mechanical inoculation (viroid co-inoculation)	Tomato, gynura, chrysanthemum	Resistance to PSTVd, CEVd and CChMVd (assessed along 20–50 dpi)	At least for 20 to 50 dpi	Carbonell et al. (2008) [40]
*CP* gene of TMV	Crude extracts of bacterially expressed dsRNA (480 bp)	300 μg of RNA per tested plant (3 μg/μL)	Mechanical inoculation (virus co-inoculation)	Tobacco	Resistance to TMV (assessed along 10–30 dpi)	More than 60 dpi	Yin et al. (2009) [46]
*CP* gene of SCMV	Crude extracts of bacterially expressed hpRNA (147 or 140 bp stem)	Serial dilutions (1 mL) of total nucleic acid 3 µg/µL	Spraying (virus co-inoculation or 1, 3, 5, 7, and 9 dpt)	Maize	Resistance to SCMV (assessed at 10, 20, 30 dpi)	At least up to 30 dpi	Gan et al. (2010) [44]
*CP* gene of PRSV	Crude extracts of bacterially expressed hpRNA (279 bp)	100 μg of hpRNA	Mechanical inoculation (virus co-inoculation or 1, 2, 3, and 5 dpt)	Papaya	Resistance to PRSV (assessed along 10–30 dpi)	More than 60 dpi	Shen et al. (2014) [45]
*CP* gene of CymMV	Crude extracts of bacterially expressed dsRNAs and ssRNAs (237 bp)	5 µg of total nucleic acid per 1 leaf (5 µg/mL)	Mechanical inoculation (virus co-inoculation)	Orchid	Resistance to CymMV (assessed at 30 dpi)	At least up to 30 dpi	Lau et al. (2014) [41]
*p126* and *CP* genes of TMV	In vitro synthesized dsRNA (p126 666 bp; CP 480 bp)	179.2 µg of *p126* and 244.8 µg of *CP* dsRNAs per plant	Mechanical inoculation (virus co-inoculation)	Tobacco	Resistance to TMV (assessed along 20 dpi)	At least up to 20 dpi	Konakalla (2016) [9]
*RP* gene of PMMoV; *2b supressor* of CMV2b	In vitro transcribed RP dsRNA (977 bp) and crude extracts of bacterially expressed 2b dsRNA (330 bp) naked or loaded into LDH	125 µL per cm^2^ (1.25 µg of dsRNA and 3.75 µg of LDH) of the leaf surface	Spraying (virus inoculation 1, 5, 20 dpt)	Tobacco, cowpea	Resistance to PMMoV and CMV (assessed at 10 dpi)	At least for 10 dpi	Mitter et al. (2017) [10]
*HC-Pro* and *CP* genes of ZYMV	In vitro synthesized dsRNAs (HC-Pro 588 bp; CP 498 bp)	40 to 60 μg of dsRNA (20 µL per leaf)	Mechanical inoculation (virus co-inoculation)	cucumber, watermelon and squash	Resistance to ZYMV (assessed along 20 dpi)	At least for 20 dpi	Kaldis et al. (2018) [11]
*RP* gene of TMV; *GFP* of TMV	Bacterially expressed or in vitro synthesized dsRNA (2 kb)	5 μg of dsRNA	Mechanical inoculation; Spraying (virus co-inoculation or 1, 2, 4, or 7 dpt)	Tobacco	Resistance to TMV (assessed at 7, 9, and 14 dpi)	At least for 14 dpi	Niehl et al. (2018) [39]
*NIb* and *CP* genes of BCMV	Chemically synthesized dsRNAs (Nib 480 bp; CP 461 bp) applied directly or loaded into LDH	100 μg of naked dsRNA (1 mL); or 250 ng of dsRNA loaded into LDH	Spraying (virus inoculation 1 or 5 dpt)	Tobacco, cowpea	Resistance to BCMV (assessed 10 and 20 dpi)	At least up to 10–20 dpi	Worrall et al. (2019) [12]

RP—replicase protein; PMMoV—pepper mild mottle virus; TEV—tobacco etch virus; AMV—alfalfa mosaic virus; dpi—days post infection; dpt—days post treatment; PSTVd—potato spindle tuber viroid; CEV—citrus exocortis viroid; CChMVd—chrysanthemum chlorotic mottle viroid; CP—coat protein; SCMV—sugarcane mosaic virus; PRSV—papaya ringspot virus; CymMV—cymbidium mosaic virus; CMV—cucumber mosaic virus; p126—TMV silencing suppressor; HC-Pro—the helper component-proteinase; LDH—layered double hydroxide clay nanosheets; ZYMV—zucchini yellow mosaic virus; GFP—green fluorescent protein; Nib—potyviral nuclear inclusion b protein; BCMV—potyvirus bean common mosaic virus.

**Table 2:** Application of external RNAs for plant insect pest resistance.

Target	RNA Type and Origin	RNA Amount	RNA Application and Feeding Assays	Plant Host	Effect	Effect Maintenance	Reference
*Cyp18A1* and *Ces* genes of BPH; *KTI* gene of ACB	In vitro synthesized dsRNA	Rice—1 mL of dsRNA (1.0 mg/mL); maize—10 mL of dsRNA (0.5 mg/mL)	Root or seed soaking; larvae feeding 24 hpt	Rice, maize	Increased insect mortality rate	At least for 3–7 dpt	Li et al. (2015) [20]
*Actin* gene of CPB	In vitro transcribed dsRNA (50, 102, 208, 266, and 297 bp)	5 μg of actin-dsRNA (200 µL) per single leaf of one plant	RNA coated over the leaf surface by the side of a 200 μL pipette tip; larvae feeding from 0.5 hpt for 7 days	potato	Lowered biological activity of CPB (monitored weight, instar stage, and mortality)	At least for 28 dpt	San Miquel and Scott (2016) [19]
*HC-Pro* gene of ZYMV	In vitro transcribed dsRNA (588 bp)	10.5 µg (10 µL) of dsRNA onto the upper side per leaflets (of a single leaf)	Mechanical inoculation (gently rubbing the surface of carborundum-dusted leaves)	tomato	dsRNA detection in tomato (local and systemic leaves) and in insects (aphids, whiteflies, and mites)	Detection at 3, 10, and 14 dpt	Gogoi et al. (2017) [21]
*JHAMT* and *Vg* genes of BMSB	In vitro synthesized dsRNA (200–500 bp)	5 µg or 20 µg in 300 µL of water (0.067 µg/µL or 0.017 µg/µL)	Immersion of the green beans in the dsRNA solution (3 h)	common bean	Decreased expression of JHAMT and Vg genes in BMSB		Ghosh et al. (2018) [22]
*AK* gene of ACP	In vitro synthesized dsRNA (200–500 bp)	200 mL of dsRNA (0.5 mg/mL);	RNA spraying;	citrange	Detection of the dsRNAs in the citrus plants; increased ACP mortality	dsRNA detection in plants 49 dpt	Ghosh et al. (2018) [22]; Hunter et al. (2012) [55]
		1 L (0.2 mg/mL), 100 mL (1.33 mg/mL), or 10 mL (1 mg/mL) of dsRNA	Soil/root drench application (soaking for 0.5 h);	citrange			
		6 mL (1.7 mg/mL) of dsRNA	trunk injections	citrange			

Cyp18A1—a cytochrome P450 enzyme; Ces—carboxylesterase; KTI—kunitz-type trypsin inhibitor; BPH—the brown planthopper (Nilaparvata lugens); ACB—Asian corn borer (Ostrinia furnacalis); CPB—Colorado potato beetle; hpt—hours post-treatment; dpt—days post-treatment; HC-Pro—the helper component-proteinase; ZYMV—zucchini yellow mosaic virus; BMSB—brown marmorated stink bug; JHAMT—juvenile hormone acid O-methyltransferase; Vg—vitellogenin; AK—arginine kinase; ACP—Asian citrus psyllid.

**Table 3:** Application of external RNAs for plant fungal resistance.

Target	RNA Treatment	RNA Amount	RNA and Fungal Application	Plant Host	Effect Assessment	Effect Maintenance	Reference
*CYP51A*, *CYP51B*, and *CYP51C* genes of *Fusarium graminearum*	In vitro synthesized CYP3-dsRNA (791 bp); siRNAs produced from dsRNA by RNAse III	10 μg dsRNA or siRNA per plate with six detached leaves (20 ng/μL in 500 µL of water)	RNA spraying; fungal inoculation 48 hpt	Barley	Inhibition of fungal growth and weaker disease symptoms; suppression of target fungal *CYP51* mRNAs	At least for 6 dpi	Koch et al. (2016) [13]
*DCL1* and *DCL2* genes of *Botrytis cinerea*	In vitro synthesized dsRNA (490 bp); siRNAs produced in vitro from the dsRNA by RNAse III	20 µL of RNA (20 ng/µL) per each plant specimen	RNA dropped onto the surface of each plant specimen; fungal inoculation or inoculation 1, 3, and 5 dpt	Tomato, strawberry, grape, lettuce, onion, rose, *Arabidopsis*	Inhibition of fungal growth and weaker disease symptoms; supression of fungal *DCL* transcripts	At least for 5 dpi	Wang et al. (2016) [14]
59 target genes of *Sclerotinia sclerotiorum*	In vitro synthesized 20 dsRNAs (200–450 bp)	10–25 μL of 200–500 ng dsRNA and 0.02–0.03% Silwet L-77	Foliar RNA application to the leaf surface with Silwet L-77; fungal inoculation after leaf drying	Oilseed rape, *Arabidopsis*	Of the 59 dsRNAs tested, 20 showed antifungal activity against *S. sclerotiorum* and *B. cinerea* and weaker disease symptoms; suppression of fungal target genes	At least for 2–4 dpi	McLoughlin et al. (2018) [15]
*Myosin 5* gene of *Fusarium asiaticum*	In vitro synthesized dsRNA (496 bp)	0.1 pM Myo5 dsRNA	RNA spraying; fungal inoculation 12 hpt	Wheat	Antifungal activity and weaker disease symptoms; reduction of fungal resistance to phenamacril fungicide; suppression of fungal *Myo5* transcript levels	Up to 7 dpi (Myo5-dsRNA); Up to 14 dpi (Myo5-dsRNA plus phenamacril)	Song et al. (2018a) [16]
*β2Tub* gene of Fusarium asiaticum	In vitro synthesized dsRNA (489 bp)	30–40 ng/µL	RNA spraying after leaf wounding with quartz sand; fungal inoculation 12 hpt	Cucumber, soya, barley, wheat	Antifungal activity against *F. asiaticum*, *B. cinerea*, *Magnaporthe oryzae*, and *Colletotrichum truncatum* and weaker disease symptoms; reduction of *F. asiaticum* resistance to carbendazim fungicide	Up to 7 dpi (β2Tub–dsRNA); up to 14 dpi (β2Tub-dsRNA plus carbendazim)	Gu et al. (2019) [18]

CYP51—cytochrome P450 lanosterol C-14 α-demethylase; dpi—days post infection; dpt—days post treatment; hpt—hours post treatment; DCL—Dicer-like protein; β2Tub—β2 –tubulin.

**Table 4:** Application of external RNAs for the suppression of plant transgenes or endogenous genes.

Target	RNA Treatment	RNA Amount	RNA Application	Plant Host	Effect Assessment	Effect Maintenance	Reference
Plant Transgenes							
*YFP* transgene	In vitro synthesized short dsRNA (21 bp) in a complex with a carrier peptide	100 µL of the RNA-peptide complex (20 pmol siRNA)	Infiltration of the complex into intact plant leaf cells using a syringe without a needle	*Arabidopsis*, poplar	Suppression of YFP protein level and fluorescence	At least for 24–36 hpt	Numata et al. (2014) [24]
*GFP* transgene	In vitro synthesized siRNAs	100 µL of aqueous siRNA solutions (10 µM)	High-pressure spraying (using a conventional compressor and an air brush pistol) at the abaxial surface of leaves	Tobacco	Local and systemic GFP fluorescence suppression (detected 2–20 dpt)	Up to 20 dpt	Dalakouras et al. (2016) [25]
*GUS* transgene	Total RNA from dsRNA-expressing bacteria (~504 bp)	100 µg of dsRNA with or without LDH	Sprayed with an atomizer	*Arabidopsis*	Reduction in GUS activity	Assessed 7 dpt	Mitter et al. (2017) [10]
*EGFP* and *NPTII* transgenes	In vitro synthesized dsRNAs (*EGFP* 720 bp; *NPTII* 599 bp)	0.35 µg/µL (100 µL per 4-week-old plant)	Spreading with sterile individual soft brushes	*Arabidopsis*	Suppression of *EGFP* and *NPTII* mRNA levels; suppression of EGFP protein level and fluorescence; induction of *EGFP* and *NPTII* DNA methylation	At least for 7–14 dpt	Dubrovina et al. (2019) [26]
**Plant Endogenous Genes**							
*EPSPS* gene	In vitro synthesized short dsRNAs (24 bp); long dsRNAs (200–250 bp)	10 µL of dsRNA on each of four leaves per plant (0.024–0.8 nM)	Leaves pre-treatment by carborundum solution or surfactant solution	Palmer Amaranth (glyphosate-tolerant)	Suppressed EPSPS transcript and protein levels; improved glyphosate efficacy	at least for 48–72 hpt	Sammons et al. (2011) [68]
*CHS* gene	In vitro synthesized short dsRNA (21 bp) in a complex with a carrier peptide	100 µL of protein carrier in a complex with the siRNA (6 pmol)	Infiltration of the complex into intact plant leaf cells using a syringe without a needle	*Arabidopsis*	Local loss of anthocyanin pigmentation	Assessed 2 dpt	Numata et al. (2014) [24]
*STM* and *WER* genes	A mixture of cationic fluorescent nanoparticles G2 and in vitro synthesized dsRNA (*STM* 450 bp; *WER* 550 bp)	1 µg of dsRNA mixed with 3 µg of gene carrier G2 per root of *Arabidopsis* once every 24 h (3 days of treatment)	By pipette	*Arabidopsis*	Suppressed transcripts of STM and WER; retarded growth and reduced meristem size; fluorescence observed throughout the root system (24 hpt)	at least for 5–7 dpt	Jiang et al. (2014) [27]
*MYB1* gene	Crude bacterial extract containing *DhMYB1* dsRNA (430 bp)	50 μL of crude bacterial extract (2 μg/μL, at 5 day intervals)	Mechanical inoculation (gently rubbing onto a flower bud using a latex-gloved finger)	hybrid orchid	Suppressed expression of DhMYB1; changed phenotype of floral cells (22, 25, and 29 dpt)	at least for 29 dpt	Lau et al. (2015) [28]
*Mob1A*, *WRKY23*, and *Actin* genes	In vitro synthesized dsRNA (*Mob1A* 554 bp; *WRKY23* 562 bp)	*Arabidopsis* and rice seeds or seedlings soaked in 0.2 or 1 mL dsRNA (1.0 mg/mL)	Root soaking	*Arabidopsis*, rice	Absorption of the dsRNA by plant roots; suppressed target genes; suppression of the root growth and seed germination; plants could not bolt or flower	at least up to 5–7 dpt	Li et al. (2015) [20]

YFP—yellow fluorescent protein; GFP—green fluorescent protein; dpt—days post treatment; hpt—hours post treatment; GUS—β-glucuronidase; EGFP—enhanced green fluorescent protein; NPTII—neomycin phosphotransferase II; EPSPS—5-enolpyruvylshikimate-3-phosphate synthase; CHS—chalcone synthase; STM—class I knotted-like homeodomain protein SHOOT MERISTEMLESS; WER—a R2R3-type MyB-related transcription factor WEREWOLF.

## Abbreviations

ACB	Asian corn borer (*Ostrinia furnacalis*)
ACP	Asian citrus psyllid
AK	Arginine kinase
AMV	Alfalfa mosaic virus
BCMV	Potyvirus bean common mosaic virus
BMSB	Brown marmorated stink bug
BPH	The brown planthopper (*Nilaparvata lugens*)
CChMVd	Chrysanthemum chlorotic mottle viroid
Ces	Carboxylesterase
CEV	Citrus exocortis viroid
CHS	Chalcone synthase
CMV	Cucumber mosaic virus
CP	Coat protein
CPB	Colorado potato beetle
CymMV	Cymbidium mosaic virus
Cyp18A1	A cytochrome P450 enzyme
CYP51	Cytochrome P450 lanosterol C-14 α-demethylase
DCL	Dicer-like protein
DICER	DICER-LIKE ribonucleases
dpi	Days post inoculation
dpt	Days post treatment
dsRNA	Double-stranded RNA
EGFP	Enhanced green fluorescent protein
EPSPS	5-Enolpyruvylshikimate-3-phosphate synthase
GFP	Green fluorescent protein
GUS	β-glucuronidase
HC-Pro	The helper component-proteinase
hpRNA	Hairpin RNAs
hpt	Hours post-treatment
JHAMT	Juvenile hormone acid O-methyltransferase
KTI	Kunitz-type trypsin inhibitor
LDH	Layered double hydroxide clay nanosheets
MPK	Mitogen activated protein kinase
Nib	Potyviral nuclear inclusion b protein
NPTII	Neomycin phosphotransferase II
p126	TMV silencing suppressor
PMMoV	Pepper mild mottle virus
PRSV	Papaya ringspot virus
PSTVd	Potato spindle tuber viroid
RISC	RNA-induced silencing complex
RdRP	RNA-dependent RNA-polymerase
ROS	Reactive oxygen species
RNAi	RNA interference or gene silencing
RP	Replicase protein
SCMV	Sugarcane mosaic virus
siRNA	Small interfering RNA
ssRNA	Single-stranded RNA
STM	Class I knotted-like homeodomain protein SHOOT MERISTEMLESS
TEV	Tobacco etch virus
β2Tub	β2–Tubulin
UTRs	5′ and 3′ Untranslated regions
Vg	Vitellogenin
WER	A R2R3-type MyB-related transcription factor WEREWOLF
YFP	Yellow fluorescent protein
ZYMV	Zucchini yellow mosaic virus
